# Functional expression of a peritrophin A-like SfPER protein is required for larval development in *Spodoptera frugiperda* (Lepidoptera: Noctuidae)

**DOI:** 10.1038/s41598-019-38734-0

**Published:** 2019-02-22

**Authors:** Claudia Rodríguez-de la Noval, Lianet Rodríguez-Cabrera, Laurent Izquierdo, Luis A. Espinosa, Daily Hernandez, Milagro Ponce, Ivis Moran-Bertot, Pilar Tellez-Rodríguez, Orlando Borras-Hidalgo, Siliang Huang, Yunchao Kan, Denis J. Wright, Camilo Ayra-Pardo

**Affiliations:** 1 Plant Division, Centre for Genetic Engineering and Biotechnology (CIGB), Havana, 10600 Cuba; 2 Analytical Unit Division, Centre for Genetic Engineering and Biotechnology (CIGB), Havana, 10600 Cuba; 3 Shandong Provincial Key Laboratory of Microbial Engineering, School of Biotechnology, Qi Lu University of Technology, Jinan, 250353 People’s Republic of China; 4 China-UK, NYNU-RRES Joint Insect Biology Laboratory, Nanyang Normal University, Henan, 473061 People’s Republic of China; 5 Department of Life Sciences, Imperial College London, Silwood Park campus, Ascot, Berkshire SL5 7PY UK; 6 Present Address: Departamento de Imunologia, Instituto de Microbiologia Professor Paulo de Góes, Universidade Federal do Rio de Janeiro, Rio de Janeiro, RJ 21941-902 Brazil

## Abstract

Peritrophins are associated with structural and functional integrity of peritrophic membranes (PM), structures composed of chitin and proteins. PM lines the insect midgut and has roles in digestion and protection from toxins. We report the full-length cDNA cloning, molecular characterization and functional analysis of SfPER, a novel PM peritrophin A protein, in *Spodoptera frugiperda*. The predicted amino acid sequence indicated SfPER’s domain structure as a CMCMC-type, consisting of a signal peptide and three chitin-binding (C) domains with two intervening mucin-like (M) domains. Phylogenetic analysis determined a close relationship between SfPER and another *S. frugiperda* PM peritrophin partial sequence. SfPER transcripts were found in larvae and adults but were absent from eggs and pupae. Chitin affinity studies with a recombinant SfPER-C1 peritrophin A-type domain fused to SUMO/His-tag confirmed that SfPER binds to chitin. Western blots of *S. frugiperda* larval proteins detected different sized variants of SfPER along the PM, with larger variants found towards the posterior PM. *In vivo* suppression of SfPER expression did not affect susceptibility of larvae to *Bacillus thuringiensis* toxin, but significantly decreased pupal weight and adult emergence, possibly due to PM structural alterations impairing digestion. Our results suggest SfPER could be a novel target for insect control.

## Introduction

The midgut of most insects is lined by a semi-permeable, peritrophic membrane (PM), a structure which optimizes insect digestion by separating ectoperitrophic processes, catalyzed by luminal digestive enzymes, from endoperitrophic processes in the space between the PM and midgut epithelial cells, catalyzed by enzymes such as luminal and microvillar aminopeptidases^1–4^. A counterflux of fluids created in the ectoperitrophic space contributes to enzymes recycling, preventing their excretion^1,4^. The PM also protects the midgut epithelium from oxidative damage^5,6^ and *Bacillus thuringiensis* (*Bt*) toxins^7^ and plant allelochemicals^8^ by excluding their absorption.

PMs can be classified into two types depending on whether they are secreted by the whole midgut epithelium (Type I) or by the cardia (Type II), an organ near the foregut-midgut junction^2,9,10^. Distension of the gut following food ingestion is the stimulus for new Type I PM formation, a process involving a midgut-specific chitin synthase B, which produces chitin precursors^11,12^, and *de novo* peritrophins secreted by midgut cells through a microapocrine mechanism^9^.

Peritrophins are integral, strongly-bound PM proteins that directly interact with the chitin fibrils scaffold through chitin-bnding domains (CBDs) and can only be released from the PM by strong denaturants^13,14^. CBDs carry multiple cysteine residues that form intra-domain and inter-molecular disulfide bridges^13,14^. Peritrophin-peritrophin interactions can also occur after CBDs interact with N-linked glycan cores attached to asparagine residues; protein multimerization increases the spatial complexity and structural stability of PM^13,14^. CBDs are classified as peritrophin A, peritrophin B, or peritrophin C domains, depending on whether they form three, four or five intra-domain disulfide bonds respectively^14^. The peritrophin A domain (PAD) is ubiquitous among insects and possess the motif CX_15–17_CX_5–6_CX_9_CX_12_CX_6–7_C. The other CBD classes are only present in dipteran larvae^14,15^. PM peritrophins may also possess one or more highly glycosylated mucin-like (MD) domains^15–17^.

The role of the PM in gut homeostasis is of particular interest and various studies have provided a rationale for the PM as a novel target site for insect control. For example, the baculovirus metalloprotease, enhancin, has been shown to degrade a highly-glycosylated structural PM protein, thereby altering PM permeability during the pathogenic process^18–20^; a *B. thuringiensis* metalloprotease (Bel) has been found to degrade a PM mucin in the cotton bollworm, *Helicoverpa armigera*, increasing the insecticidal potency of Cry1Ac toxin^21^; and chemical dissociation of peritrophins from chitin fibrils has been shown to disrupt PM formation, and increase susceptibility of insects to baculovirus infection and bacterial toxins^22,23^. In addition, an inducible cysteine protease (Mir1) from *Zea mays* has been shown to increase PM permeabilization and reduce larval growth in *Spodoptera frugiperda*, a major pest of maize in the Americas and a recent invasive species in Africa and southern India^24–27^. Resistance of Mir1-overexpressing plants to *S. frugiperda* demonstrates the potential of the PM as a target for pest control strategies.

RNA interference (RNAi)^28^ has proved successful in insects and has been used to generate transgenic plants expressing a dsRNA directed against suitable insect target genes^29–32^. Since the insect midgut is in close proximity to the site of dsRNA entry, the potential of midgut genes as targets for RNAi has also been investigated, including genes involved in PM synthesis^33–39^.

In the present work, we cloned and characterized the full-length cDNA of a new PM protein from *S. frugiperda*, referred to as SfPER. This protein possesses three PAD-type CBDs with two intervening MDs, and its chitin-binding activity has been verified. Transcriptional profiling displayed SfPER expression pattern in the different life-cycle stages of *S frugiperda*. Immunoblotting experiments on larval proteins showed SfPER distribution in the PM. RNAi-mediated gene expression suppression revealed the importance of PM SfPER synthesis for larval development in *S. frugiperda*.

## Results

### Cloning of a new *S. frugiperda* gene and sequence analysis

**Figure 1:**
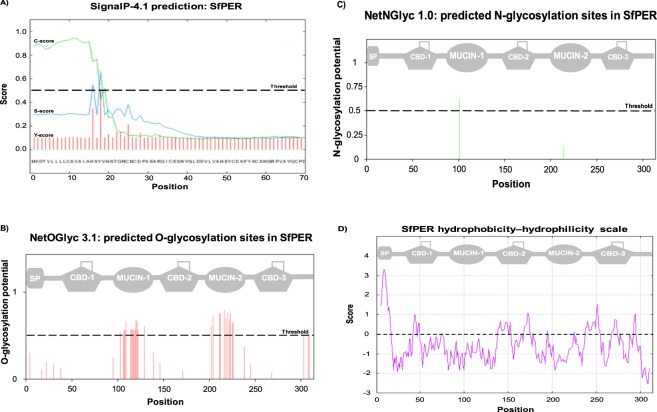
SfPER amino acid sequence analysis. (**A**) Prediction of SfPER signal peptide (SP) M_1_KDTVLLLLCAVALAHS_17_ and SP cleavage site location between S_17_ and Y_18_ residues in the sequence AHS_17_-Y_18_V (D = 0.782 D-cutoff = 0.450), as given by the combination of C-score, S-score and Y-score artificial neural networks in SignalP 4.1 server. (**B**) Prediction of O-glycosylation sites in SfPER by the NetOGlyc 3.1 program. The calculated G-score for multiple serine and threonine residues in SfPER sequence is indicated by vertical lines, with those crossing the default threshold of 0.5 as potential for O-glycosylation. (**C**) Prediction of N-glycosylation sites in SfPER by the NetNGlyc 1.0 program. The SfPER N_101_ residue in ‘N_101_GTD’ sequon showed a ‘potential’ score crossing the default threshold of 0.5 that represents a predicted glycosylated site. The ‘potential’ score is the averaged output of nine neural networks. (**D**) SfPER hydrophobicity-hydrophilicity plot according to Kyte & Doolittle^40^, where scores <0 are increasingly hydrophilic and >0 are increasingly hydrophobic. The distribution of SP, CBD (chitin-binding domains) and MUCIN (mucin-like domains) along the SfPER amino acid sequence is depicted at the top of the graph in panels B, C and D.

The full-length cDNA sequence of SfPER was obtained using the Rapid Amplification of cDNA Ends (RACE) procedure and analysed. The cDNA (GenBank acc. no. MG786480) was 1120 base pairs (bp) in length and contained a 945 bp open reading frame (ORF) spanning nucleotides (nt) 62 to 1006. The ORF encoded a protein consisting of 314 amino acids, which included a 17-residue signal peptide (Fig. 1A), with a calculated molecular mass for the mature protein of 32.2 kDa and an isoelectric point (pI) of 4.43. The 5′ untranslated region (UTR) and 3′ UTR of SfPER cDNA were 61 and 114 bp, respectively.

Prediction of the SfPER domain structure by SMART revealed the existence of three CBDs, spanning amino acids 33–91 (E-value = 3.34e-15), 138–195 (E-value = 3.39e-16) and 237–294 (E-value = 6.83e-12), respectively. The spacer elements separating CBD1 from CBD2 and CBD2 from CBD3 were found to concentrate 69% (27 of 39) of serine plus threonine residues of the mature protein, with 96% (26 of 27) of them predicted as mucin-type O-linked glycosylation sites (Fig. 1B). Accordingly, SfPER was classified as a peritrophin of CMCMC type consisting of three CBDs with two intervening MDs domains. Additionally, one putative N-linked glycosylation site was predicted at the asparagine Asn_101_ residue (Fig. 1C). The hydropathic analysis of mature SfPER protein yielded hydrophobic patches were concentrated on the three CBDs, whereas, the MDs were hydrophilic (Fig. 1D). The calculation of ‘Grand Average of Hydropathy’ (GRAVY) values for each CBD separately indicated CBD1 (−0.637) to be much less hydrophobic than CBD2 (−0.229) or CBD3 (−0.153)^40^.

**Figure 2:**
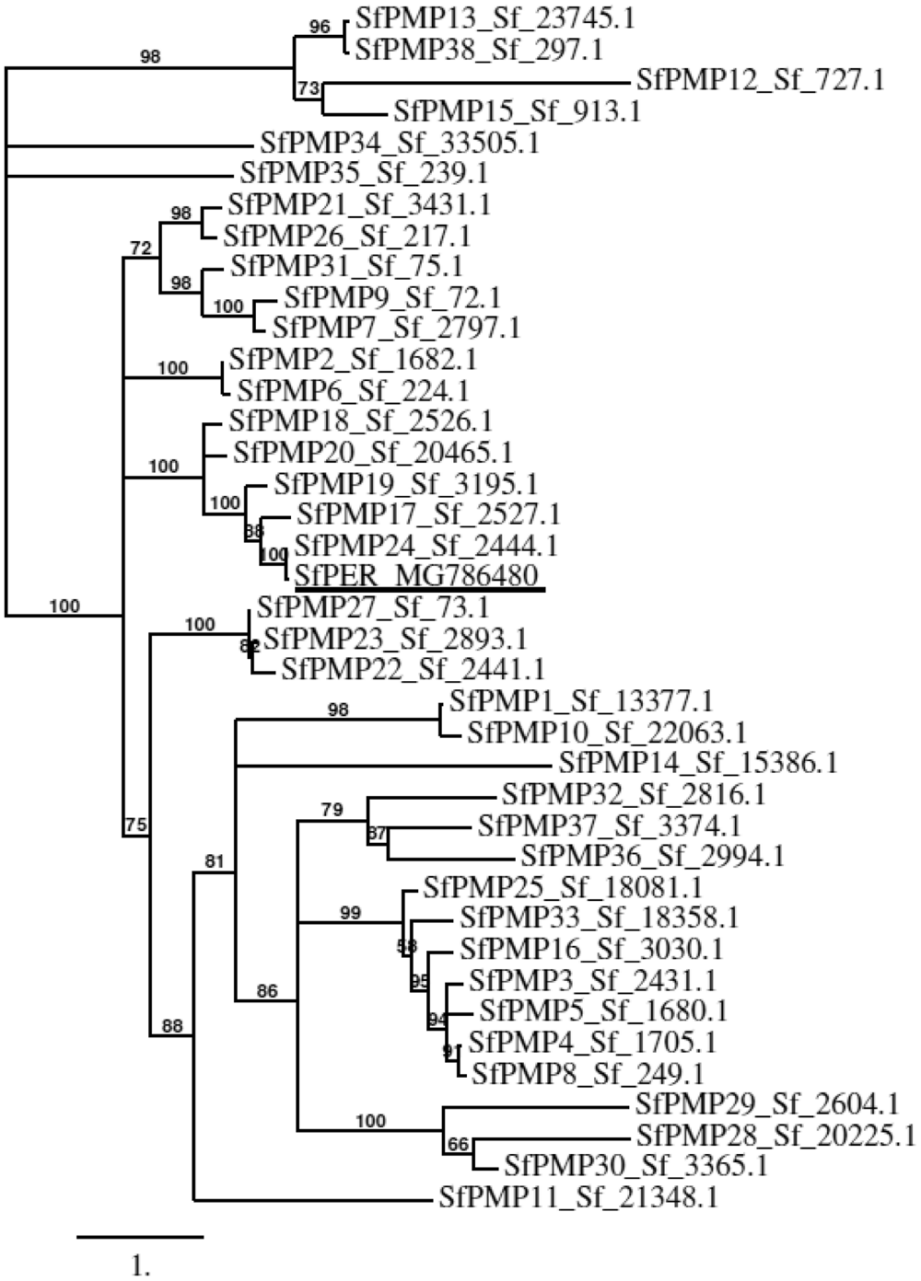
Phylogenetic analysis of SfPER. A phylogram showing the relationship between *S. frugiperda* SfPER (MG786480) and 38 complete and partial amino acid sequences corresponding to PM peritrophins identified in a *S. frugiperda* transcriptome^41^ as obtained using PhyML and TreeDyn programs. SfPER is underlined. Deduced amino acid sequence for SfPER protein and sequences encoded by related genes were phylogenetically aligned using the Muscle vs. 3.7 program. Bootstrap values greater than 50 are indicated along branches. The scale bar indicates the evolutionary distance.

Multiple alignment and phylogenetic relationship analysis of the predicted amino acids sequence for mature SfPER protein with 38 *S. frugiperda* PM polypeptides (SfPMP) deducted from transcriptomic data^41^ yielded 100% identity with a partial sequence denoted SfPMP24 (ID: Sf_2444.1) (Fig. 2). Specific alignment between SfPER and SfPMP24 showed the latter to be most likely SfPER carboxyl-terminus sequence, lacking the first 144 amino acids comprising the signal peptide, CBD1 and MD1 (Supplementary Fig. 1).

### SfPER is expressed during the insect feeding stages

**Figure 3:**
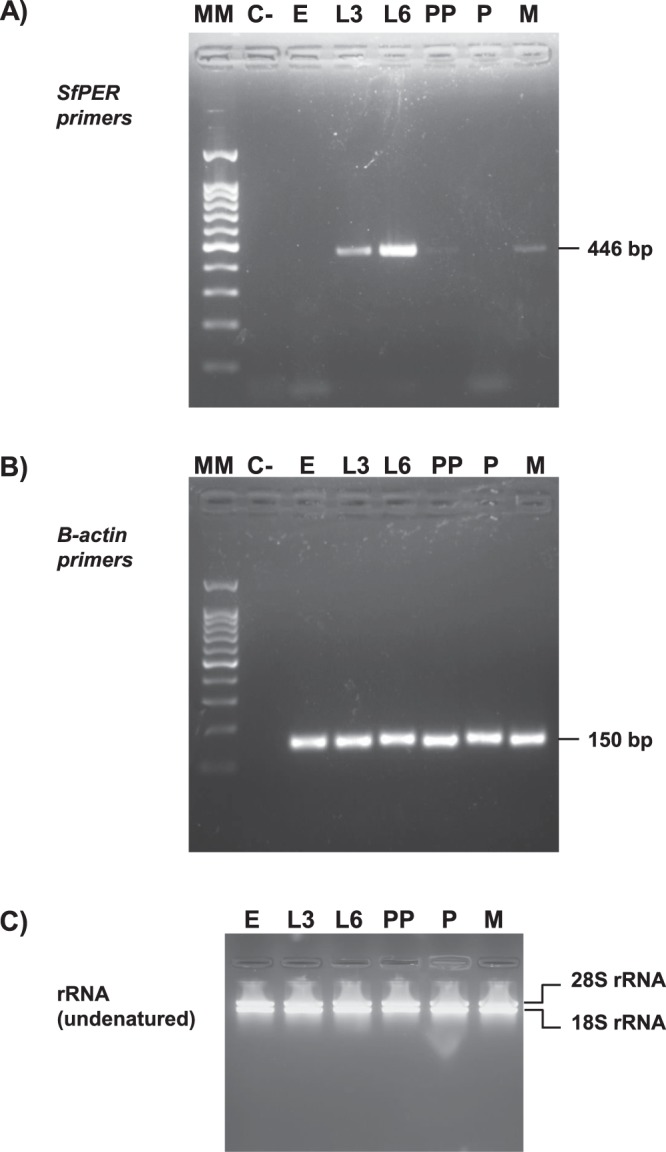
SfPER expression analysis. (**A**) *SfPER* mRNA detection at different stages of the *S. frugiperda* life cycle by RT-PCR. (**B**) Amplification of a fragment from the constitutive *β-actin* gene used as control. (**C**) Total RNA preparations. rRNA (undenatured) refers to 28S and 18S ribosomal RNAs prior heat-denaturation. C-, no template control; E, embryo; L3, third larval instar; L6, sixth (last) larval instar; PP, pre-pupae; P, pupae; M, moths (adults); MM, 100 bp DNA ladder.

To determine whether there were any developmental stage-specific differences in expression, the *SfPER* mRNA accumulation in eggs, larvae, pupae and adults was explored by RT-PCR (Fig. 3). In *S. frugiperda*, the larva stage goes through six different instars each varying in size and pattern before metamorphosing into a pupa^26^. Therefore, we compared midgut SfPER expression between young and old larvae by using insects from third and sixth (last) instars (neonate-first and second larval instars were too small for midgut dissection). We also examined pre-pupae, a quiescent stage that occurs prior to pupal formation, where the PM in lepidopteran insects is reported to be degraded^42^. *SfPER* transcripts were detected during the actively feeding stages of the insect life cycle (larvae and adult) and were absent from eggs and pupae (Fig. 3A), a pattern that is compatible with SfPER having a role in digestion and/or absorption of nutrients. In the larval stages, *SfPER* mRNA levels progressively increased from the third to sixth larval instars. The accumulation rate of *SfPER* was found to dramatically decrease during the progression from sixth-instar larvae to the pre-pupal stage.

### SfPER CBDs are PAD sequences and bind chitin

In order to characterize the SfPER protein, we attempted to produce the recombinant product in an *Escherichia coli* host. However, no product could be detected in the supernatant or cell debris of induced cultures carrying a plasmid with either 6xHis-SfPER or 6xHis-SUMO/SfPER fusions (Data not shown). Predicted SfPER features such as acidic pI and low protein stability (IIS = 49.8, see Methods) are known to increase degradation of recombinant polypeptides^43^ and could be responsible for the lack of expression of the full-length protein in *E. coli*. Structural PMPs use CBDs to link chitin fibrils in the PM and we therefore cloned one of the three SfPER CBDs (SfPER-C1) into the SUMO/His-tag system to produce the fusion protein 6xHis-SUMO/SfPER-C1. CBD1 was chosen among the three SfPER CBDs because of its lower hydrophobicity.

**Figure 4:**
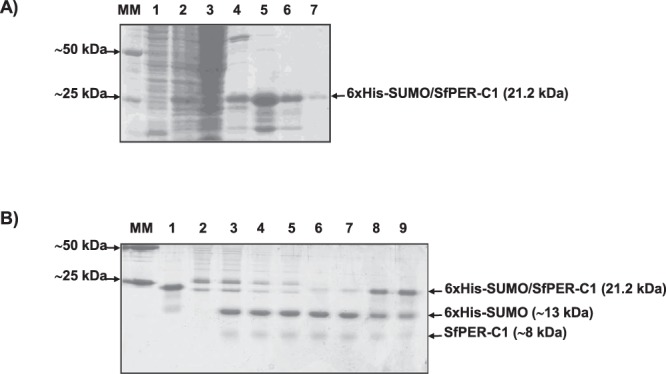
Obtainment of a recombinant SfPER-C1 domain in the SUMO/His-tag fusion system. (**A**) SDS-12% PAGE analysis of recombinant 6xHis-SUMO/SfPER-C1 expression and purification process, lane 1: supernatant sample from uninduced BL21 (DE3) cells, lane 2: supernatant sample 4 h post-induction with IPTG, lane 3: sample from nickel column flow through peak, lane 4: sample from nickel column wash peak (50 mM imidazole), lane 5: sample from elution peak with 100 mM imidazole, lane 6: sample from elution peak with 200 mM imidazole, lane 7: sample from elution peak with 300 mM imidazole. MM: protein molecular marker. (**B**) Tricine SDS-12.5% PAGE analysis of 6xHis-SUMO/SfPER-C1 cleavage reactions with different Ulp1 concentrations. Lane 1: purified recombinant 6xHis-SUMO/SfPER-C1, lane 2: Ulp1 protease (10 U), lanes 3–9: products of 6xHis-SUMO/SfPER-C1 digestion with different Ulp1 concentrations ranging 10 U–0.15 U. MM: protein molecular marker.

The recombinant expression and subsequent nickel-affinity purification of 6xHis-SUMO/SfPER-C1 was pursued by SDS-12% PAGE (Fig. 4A). A product of ∼21 kDa, the predicted size for 6xHis-SUMO/SfPER-C1, was visible in the cell supernatant at 4 h post-induction with isopropyl β-D-1-thiogalactopyranoside (IPTG) (Fig. 4A; lane 2). Proteins without 6× His tags and not firmly bound to the nickel resin were removed after column washing with a buffer containing 20 mM imidazole. The elution of a ∼21 kDa protein was obtained with 75, 150 and 300 mM of imidazole, with most of the protein eluting in the lowest concentration (Fig. 4A; lanes 5, 6 and 7). Low molecular mass contaminants co-eluted with the ∼21 kDa protein at all imidazole concentrations. These contaminants could represent partially carboxyl-terminus degraded 6xHis-SUMO/SfPER-C1 forms.

To obtain pure SfPER-C1 protein, the 6xHis-SUMO was cleaved from 6xHis-SUMO/SfPER-C1 with SUMO protease 1 (Ulp1) (Fig. 4B). The dialyzed protein was incubated with Ulp1 as described in the Methods to determine the optimal enzyme concentration; i.e., the greatest enzyme dilution (in a serial dilution) that still totally cleaves the fusion protein into SUMO/His-tag and target proteins. In the enzymatic reaction, Ulp1 concentrations above 2.5 U cleaved 6xHis-SUMO/SfPER-C1, which was shown on a Tricine SDS-12.5% PAGE gel as a reduction in the intensity of the band corresponding to the fusion protein (∼21 kDa) and the appearance of two new bands of lower size i.e. 6xHis-SUMO (∼13 kDa) and SfPER-C1 (∼8 kDa) (Fig. 4B, lanes 3, 4 and 5). SfPER-C1 was highly unstable in solution and degraded before it could be purified from the components bearing His tags, such as SUMO and Ulp1 (Data not shown).

Mass spectrometric analysis of purified 6xHis-SUMO/SfPER-C1 product covered 87% of the amino acids sequence and identified it as such, with an average molecular weight of 21.2 kDa. ESI-MS/MS spectra of QICESNYGLDDVLVAHEYCDK (m = 804.69), CANGRPVAYQCPDNLLYDPVAER (m = 854.73) and CEWPNEVNCGNRPI (m = 814.85) peptides, generated from trypsin-digested 6xHis-SUMO/SfPER-C1, identified three intra-domain disulfide bonds that corresponded with Cys_36_-Cys_52_, Cys_58_-Cys_68_ and Cys_81_-Cys_89_ residues from CBD1 in SfPER primary sequence, as expected for a characteristic PAD arrangement in SfPER-C1. Multiple sequence alignment of SfPER-C1 with the other two CBDs of SfPER showed the consensus arrangement of PAD in all cases, including the six conserved cysteines residues involved in intra-domain disulfide bonds. (Supplementary Fig. 2).

**Figure 5:**
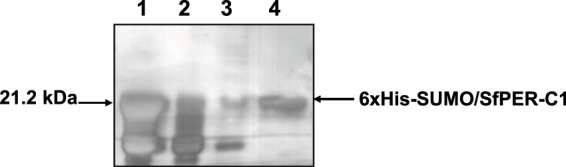
Chitin affinity chromatography of 6xHis-SUMO/SfPER-C1. SDS-12% PAGE analysis of 6xHis-SUMO/SfPER-C1 chitin-affinity purification process. Lane 1: 6xHis-SUMO/SfPER-C1 protein from metal affinity purification process applied onto the chitin resin, lane 2: sample from chitin column flow through peak, lane 3: sample from chitin column wash peak with PBS 1X, lane 4: sample from elution peak with 5% SDS.

Predicted chitin-binding properties of the SfPER-C1 PAD were verified by further purification of the recombinant fusion protein through a chitin column (Fig. 5). Low molecular weight contaminants from nickel-affinity purification step did not bind to the chitin resin and were almost totally removed during the column washing step with PBS; 6xHis-SUMO/SfPER-C1 was then eluted with PBS plus 5% SDS. The purity for the recombinant protein exceeded 95% according to SDS-12% PAGE analysis (Fig. 5, lane 4).

### SfPER is expressed in the PM

**Figure 6:**
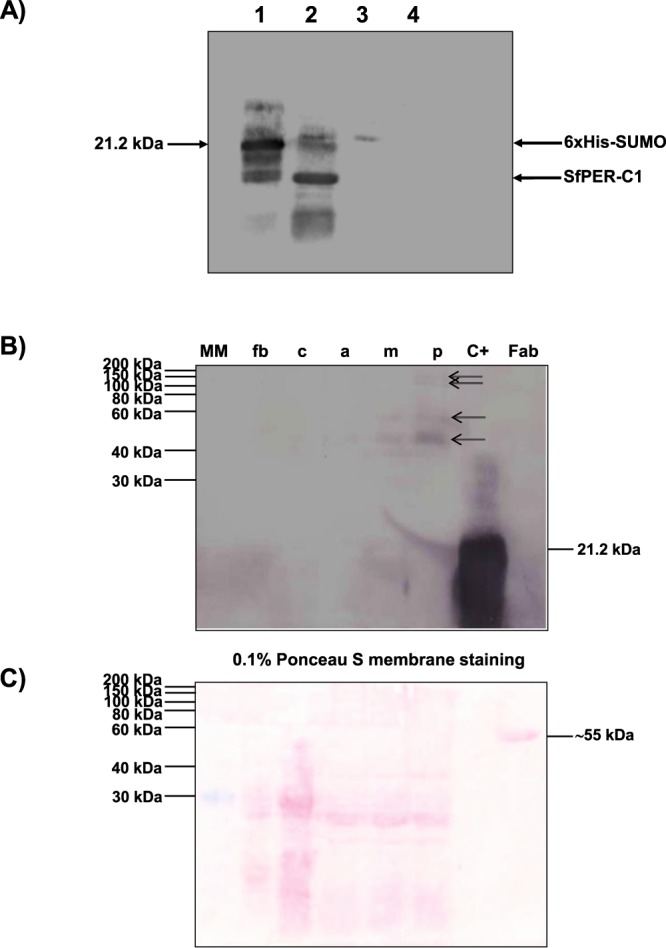
Western blotting of SfPER using rabbit polyclonal anti-6xHis-SUMO/SfPER-C1 antibodies. (**A**) Detection of anti-SfPER-C1 antibody species. Lane 1: purified recombinant 6xHis-SUMO/SfPER-C1 (1 μg), lane 2: products of 6xHis-SUMO/SfPER-C1 digestion with Ulp1 protease, lane 3: Ulp1 protease, lane 4: BSA. (**B**) Immunodetection of SfPER in larval tissues. Twenty micrograms of larval proteins extracted from fat body (fb), cuticle (c), anterior PM (a), middle PM (m) and posterior PM (p) were loaded into SDS-12% PAGE under non-reducing conditions. Purified recombinant 6xHis-SUMO/SfPER-C1 (10 ng) was used as control. Fab: goat Fab fragment anti-rabbit IgG, non-reduced. MM: wide-range (30–200 kDa) unstained protein weight marker. Electrophoresed proteins were blotted onto nitrocellulose membranes and then probed with polyclonal anti-6xHis-SUMO/SfPER-C1 antibodies. Arrows indicate SfPER variants. The working dilution of polyclonal anti-6xHis-SUMO/SfPER-C1 antibody was 1:25 in TBS-T (TBS containing 0.2% Tween 20). A commercial monoclonal alkaline phosphatase (AP)-labeled goat anti-rabbit IgG (1:5000 in TBS-T) was used for detection. Blots were developed with 5-bromo-4-chloro-3-indolyl phosphate (BCIP)/nitro blue tetrazolium (NBT) insoluble substrate in developing buffer. (**C**) 0.1% Ponceau S reversible staining of the larval proteins’ blot to determine transfer efficiency.

To detect *SfPER* expression in different *S. frugiperda* larval tissues, a rabbit polyclonal anti-6xHis-SUMO/SfPER-C1 antibody was produced using purified 6xHis-SUMO/SfPER-C1 as antigen. First, we verified the existence of antibody species recognizing epitopes on SfPER-C1 by Western blotting of 6xHis-SUMO/SfPER-C1 Ulp1-cleavage products (Fig. 6A). Immunoblotting experiments showed polyclonal antibodies not only detected the fusion protein (Fig. 6A, lane 1) but also each of the products resulting from its digestion with Ulp1 protease i.e., SUMO/His-tag and SfPER-C1 (Fig. 6A, lane 2). In contrast, no recognition of nonspecific Ulp1 and BSA proteins was detected (Fig. 6A, lanes 3 and 4).

Western blotting of insect proteins separated in SDS-12% PAGE under non-reducing conditions detected at least four different sized variants of SfPER along the PM (anterior, middle and posterior sections); whereas no signal was produced for cuticle and fat body samples (Fig. 6B). The molecular weights of the SfPER variants ranged between 40 and 150 kDa, which are above the predicted size of the mature SfPER i.e. ∼32.2 kDa, and probably correspond to protein glycoforms due to the existence of N- and O-glycosylation sites in SfPER. Interestingly, the largest SfPER variants were detected toward the posterior PM, where the PM’s structure becomes more elaborated.

### SfPER expression suppression affects insect performance

**Figure 7:**
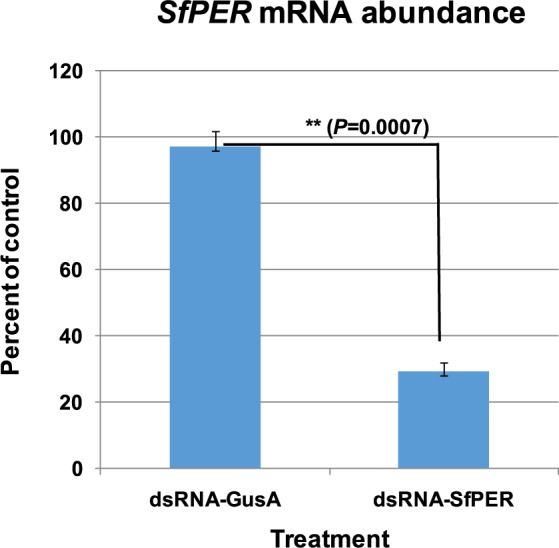
Reduction of *SfPER* expression in the larval midgut after RNA interference silencing. Transcription of *SfPER* was monitored by One-step real-timeRT-PCR in the midgut cells of third-instar larvae 48 h after droplet feeding with delivery buffer (DB) solutions containing 2.5 μg of double-stranded RNA (dsRNA) *per* larva from either dsRNA-SfPER or bacterial (nonspecific) dsRNA-GusA from the *Escherichia coli β-D-glucuronidase* gene. The results are expressed as the percent of untreated control (DB only) after normalization with the reference *β-actin* gene. The experiments were performed twice. Bars represent means of two independent replicates ±SE and were compared by *t*-test (*P* < 0.05).

We conducted RNAi-mediated expression suppression of *SfPER* in order to evaluate the importance of this PM peritrophin in the response to a bacterial toxin and for insect development. Third-instar larvae were fed with specific double-stranded RNA (dsRNA-SfPER), with larvae fed with non-specific bacterial dsRNA from the *E. coli β-D-glucuronidase* gene (dsRNA-GusA) or with buffer only as controls. *SfPER* mRNA levels in the midgut tissue 48 h after droplet feeding with dsRNA-SfPER were found to be almost 70% lower compared with dsRNA-GusA or buffer-fed (mock) control insects (Fig. 7).

**Figure 8:**
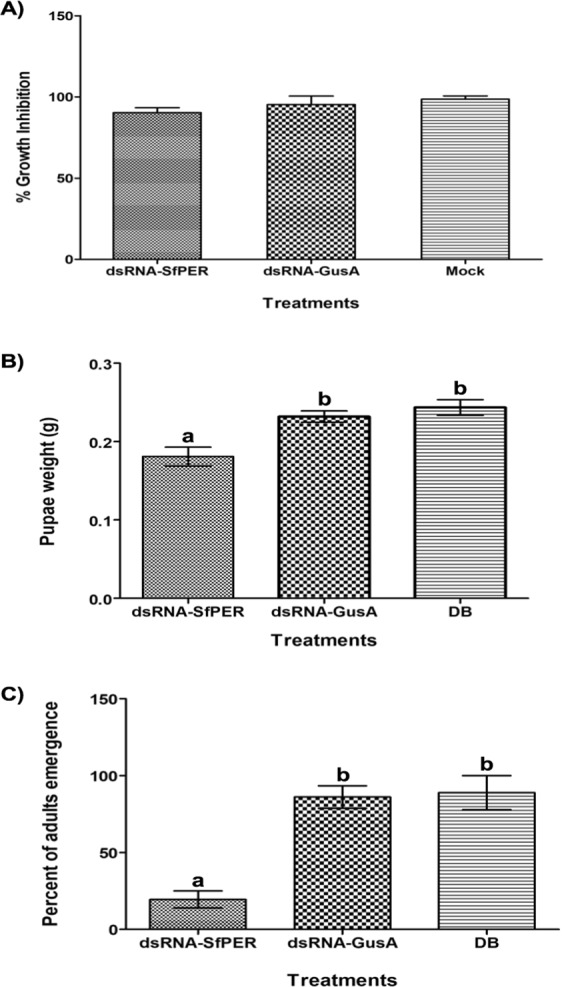
Effects of RNA interference-mediated *SfPER* expression suppression on *S. frugiperda* performance. Insects treated with dsRNA (dsRNA-*SfPER* and dsRNA-GusA) and Mock (delivery buffer) were compared in terms of (**A**) percent of growth inhibition (%GI) caused by the exposure to 2 μg/cm^2^ of Bt Cry1Ca1 protoxin, (**B**) pupae weight, and (**C**) percent of adult emergence. Bars represent means of two independent replicates ±SE. Different letters indicate significant differences (*P* < 0.05).

Following RNAi, pre-weighed larvae were exposed to diet with 2 μg/cm^2^ of Bt Cry1Ca1 protoxin and re-weighed after 48 h exposure to toxin. The Bt Cry1Ca1 protoxin caused a growth inhibition of almost 100% for all treatments including the controls (Fig. 8A). However, while RNAi *per se* did not affect larval growth or cause larval mortality, other features of insect development were found to be compromised in the group of insects fed with dsRNA-SfPER. A lag of 7 ± 2 days in the time of appearance of the first pupae was seen in insects in which *SfPER* had been suppressed compared with the control treatments. The dsRNA-SfPER treatment also caused a significant decrease in pupal weight and adult emergence compared with the controls, which did not show significant differences between them (Fig. 8B,C).

## Discussion

In the present study, we have cloned and characterized the full-length cDNA sequence of SfPER, a new CMCMC-type peritrophin A-like PM protein from *S. frugiperda*. Phylogenetic analysis found SfPER to be related to the 38 SfPMPs previously identified in *S. frugiperda*, of which only 15 have a complete sequence verified with domains structure elucidated^41^. In the phylogram, SfPER clustered at 100% bootstrap level with SfPMP24, a sequence encoding the C-terminal fragment of a PM peritrophin, which suggests SfPER is most likely the full-length product. Pairwise alignment between these two amino acids sequences found SfPMP24 to be identical to SfPER apart from four amino acids residues and that SfPER is 144 amino acids longer at the N-terminal region, which comprises the signal peptides, CBD1 and MD1. Interestingly, the SfPER domain structure, which consists of a triple CBD arrangement with two interspersed MDs, was not present in any of the 15 full-length SfPMPs reported by Dias *et al*.^41^.

We detected *SfPER* transcripts in midgut samples from the larval and adult stages of the *S. frugiperda* life cycle, with *SfPER* mRNA levels increasing considerably from L3 to L6 larval instars before dropping to almost undetectable levels in pre-pupae. As a PM structural protein, *SfPER* gene expression regulation is expected to be tightly correlated with PM synthesis. In lepidopteran insects, PM is produced in the midgut during actively feeding stages of the life cycle, i.e. larvae and adults, to facilitate digestion among other functions^1–4^. PM degradation is triggered during larval molts and larval-pupal transformation, which are characterized by insect feeding cessation^2,42^. PMP downregulation during metamorphosis has been recently associated with 20-hydroxyecdysone (20E) titers rather than with cessation of insect feeding per se. Chen *et al*.^42^ found mRNA levels of two peritrophin-like proteins from the common cutworm, *Spodoptera litura* were suppressed after injection of 20E into final instar larvae, whereas, constitutive expression of these proteins remained unaltered under starvation conditions. A binding site for a 20E-responding factor Brc-Z was predicted in the 5′ upstream region of one of the cloned *S. litura* PMPs^42^. The cloning and analysis of 5′ upstream region of *SfPER* gene would give more information about expression regulation of this new *S. frugiperda* PMP. A few midgut peritrophins are continuously produced, even in the absence of PM^44^, which might suggest a role for them other than structural constituents of PM.

In the present study, data from SfPER-C1 ESI-MS/MS analysis and “in silico” SfPER CBD sequences alignment have indicated SfPER CBDs adopt a typical PAD arrangement, which includes the three intra-domain disulfide bonds between the six conserved cysteines residues that configure this region into a compact domain. The involvement of PAD disulphide bonds in proteolytic resistance of PMPs against the hydrolytic enzymes present in the insect gut lumen has been demonstrated for *Trichoplusia ni* IIM protein^16^; treatment of IIM with the disulfide bond-reducing agent dithiothreitol causing its degradation by endogenous digestive proteases^17^. In our study, a purified SUMO fusion protein containing the first of three SfPER PAD sequences (SfPER-C1) bound to a commercial chitin resin, implying PADs confer SfPER affinity for chitin within the PM. Thus, the triple-PAD structure of SfPER may allow each protein to bind three adjacent chitin polymer chains, contributing strength and elasticity, and determining the porosity of the *S. frugiperda* PM. A five-PAD PMP TcPMP5-B was found to be required for the maintenance of PM integrity and barrier functions in the beetle *Tribolium castaneum*, as depletion of its mRNA via RNA interference increased PM porosity^38^.

Our Western blot experiments detected variants size of SfPER in the PM of *S. frugiperda* larvae, whereas, no expression was found in the cuticle or fat body, tissues that also express proteins with PADs^45–47^. We speculate the different sized variants of SfPER are N- and O-glycoforms since such post-translational modifications increase the molecular weight of proteins and generate diversity^48^. Also, N- and O-glycosylations show site-specific micro-heterogeneity, turning a single isolated protein into a very complex mixture of glycosylated species that may exhibit different locations, functions and lifespan^49^. In the present study, the different sized variants of SfPER were found to be expressed by different PM sections, with the larger variants toward the posterior PM. The gene expression rate along the midgut of insects is not homogeneous^38,41^ and this could also affect enzymes involved in protein glycosylation. Interestingly, the PM is less organized in the anterior region of insects compared with their posterior region. While the anterior part is a gel-like structure, the posterior part is thicker, mechanically stronger and less permeable to larger molecules^1,2,10,44^. The latter could suggest a direct link between the size of a SfPER variant and PM permeability. A potential role of PMP glycosylation in the control of PM pore diameter and PM hydration has been recently suggested^38^. In this regard, several insect species have been shown to contain PMPs with intervening O-glycosylated mucins^2,4,15,16,38^. A combined study of glycosyltransferase gene expression profiles and glycomics at each midgut region would draw more elements to explain the role of different sized variants of SfPER in PM function.

The relatively large size of some SfPER variants on non-reducing SDS-PAGE might also correspond with protein oligomers. Additional (non-PAD) cysteine residues present in SfPER may be involved in intermolecular interactions leading to mucin multimerization as commonly found in vertebrate mucins^2^. However, we could not prove this hypothesis since the polyclonal anti-6xHis-SUMO/SfPER-C1 antibody did not bind to SfPER on reducing SDS-PAGE (Data not shown).

The RNAi of SfPER experiments with *S. frugiperda* showed a significant decrease in both pupal weight and percent adult emergence but not on larval growth. This is intriguing since the *SfPER* expression pattern did not suggest a role for this protein during metamorphosis but rather in the larval midgut. We speculate that the lack of immediate effects of RNAi on larval growth could be mainly explained by a study which proposed midgut epithelial cells store enough PM proteins in secretory vesicles to be secreted into the midgut lumen after each meal^50^. In addition, a combination of the 30% of SfPER transcription remaining in knockdown insects observed in the present study and a low turnover rate for the N- and O-glycosylated SfPER protein could contribute to delaying the observed phenotypic effects of RNAi.

The decrease in pupal weight and adult emergence in dsRNA-SfPER treated *S. frugiperda* is most likely to be due to alterations in PM functions related to digestion and nutrition since development of lepidopteran insects depends on the quality of the food consumed during larval stages^51^. For instance, final instar caterpillars of *Spodoptera littoralis* fed with a low quality protein (zein) showed a delayed development and reduced pupal mass when low P:C (protein:carbohydrate) ratio diets were used^52^. Such a performance decrease was likely due to a deficiency of essential amino acids in the food^53,54^ that may incur fitness costs^55^. In general, PM degradation has been shown to directly impact on insect digestion. For example, *Bombyx mori* larvae fed with a sublethal concentration of chitinase showed a disrupted PM and impaired nutritional performance due to an increase in the metabolic cost associated with the conversion of food into body mass^56^. Other evidence for the importance of the PM for normal growth is based on the action of PM disrupting factors, such as calcofluor white, proteases and lectins^7,17,18,21,57^. In the one previous report that examined the role of PMPs in the maintenance of PM functional integrity, RNAi-mediated depletion of two *T. castaneum* PMPs resulted in loss of fat body reserves, which affected molting and caused insect death at the larval-pupal molt and at the pharate adult stage^38^.

The present study is the first to functionally link a PMP with insect development in Lepidoptera. There is also the possibility of additional functions for SfPER unrelated to nutrition. The *S. frugriperda* expressed sequence tag fragment ESTSFRC3 (GenBank accession no. EL618681) used in this study to clone the full-length cDNA of *SfPER*, was previously identified by our group among the insect midgut genes regulated during *Bt* pathogenesis^58^. Future experiments could also target younger larval instars, where the gene-specific transcription rate is lower and *SfPER* mRNA can be more efficiently depleted by RNAi. The low affinity (signal) displayed by the polyclonal anti-SfPER-1C antibodies used against SfPER in PM samples made it impractical to detect the effects of RNAi on SfPER at the protein level. The heterologous expression of *SfPER* full-length cDNA sequence in a more appropriated host system such as insect cells could help protein characterization and yield a better antigen for production of high-quality polyclonal or monoclonal antibodies to be used in immunodetection studies with *S. frugiperda* PM samples.

**Figure 9:**
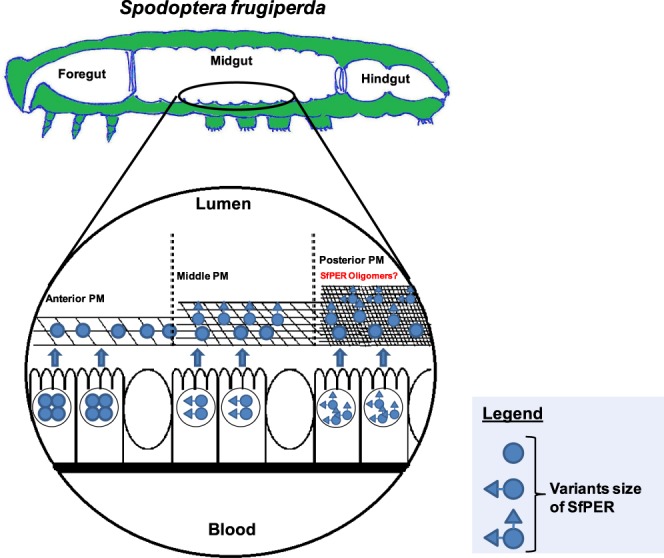
Schematic illustration of a model for SfPER functions in the *S. frugiperda* peritrophic membrane. This drawing was created by contributing author C.A.-P.

In conclusion, we have demonstrated that SfPER is a new PM CMCMC-type peritrophin A-like protein from *S. frugiperda*, whose reduction using RNAi decreases pupae weight and adult emergence, two important parameters of insect development and fitness. Using our data together with data from the literature, we propose a model for SfPER function as a molecular linker of chitin fibrils and bundles in the *S. frugiperda* PM, which would eventually regulate PM’ function associated to digestion and nutrition (Fig. 9). In our model, *SfPER* is constitutively expressed along the length of the midgut, with transcription occurring mainly in the anterior midgut^41^. N-linked/O-linked glycosylations in SfPER can generate different sized variants and glycoforms that are expressed by different midgut regions with larger ones accumulating in the posterior midgut. SfPER variants are synthesized and stored in secretory vesicles^50^ before secretion to the endoperitrophic space through a microapocrine mechanism^9^. Once in the PM, SfPER binds to newly synthesized chitin fibrils through its three PAD-type CBDs. SfPER multimers could be formed toward the posterior PM through the interaction between non-PAD cysteine residues or between a CBD from one molecule and the N-linked glycan core at Asn_101_ residue of another, which increase further the PM spatial complexity, although their existence was not proved here. It is clear that alterations of SfPER content have consequences on PM functions, eventually affecting digestion efficiency. We therefore consider SfPER to be a potential target for novel pest-control strategies in *S. frugiperda*.

## Methods

### Insects

A *S. frugiperda* population was maintained in the CIGB (Havana, Cuba) at 28 ± 2 °C, ca. 80% relative humidity and 16:8 h (light: dark) cycle on artificial diet (Frontier Agricultural Sciences).

### Total RNA extraction

Insects were pre-chilled on ice for dissection. Total RNA from samples of eggs, neonates, larval (L3 and L6) midgut, pre-pupae, pupae and adult abdomen was isolated with the SV total RNA isolation system (Promega). RNA quality and integrity was verified by 1% agarose gel electrophoresis. RNA concentration was measured using a NanoDrop 2000 Spectrophotometer (Thermo Scientific).

### Cloning of novel *S. frugiperda* gene

The nucleotide sequence of *S. frugiperda* expressed sequence tag fragment ESTSFRC3 (GenBank accession no. EL618681) with homology to a peritrophin A-like gene, was used for cloning the full-length cDNA of *SfPER* gene from midgut total RNA with the aid of the BD SMART RACE cDNA Amplification Kit (Clontech), following the protocol provided by the manufacturer. Complementary gene-specific primers (RACE-5′) 5′-ccgcagacgacattctcgggccagtc-3′ and (RACE-3′) 5′-gactggcccgagaatgtcgtctgcgg-3′ were designed based on the ESTSFRC3 nucleotide sequence and used for PCR amplify 5′- and 3′-RACE fragments, respectively.

### SfPER DNA and protein sequence analysis

Gene assembly, translation of nucleotide sequence and open reading frame identification were accomplished with ApE “A plasmid Editor” (http://biologylabs.utah.edu/jorgensen/wayned/ape/). Protein sequence analysis tools used included: “ProtParam” (https://web.expasy.org/protparam/) for calculation of theoretical isoelectric point and molecular weight, amino acid composition, instability index score (IIS; stable proteins show IIS <40), and grand average of hydropathicity (GRAVY), “SignalP 4.1 Server” (http://www.cbs.dtu.dk/services/SignalP/)^59^ for signal peptide prediction, “SMART” (http://smart.embl-heidelberg.de) for protein domains identification, “NetNGlyc 1.0” (http://www.cbs.dtu.dk/services/NetNGlyc/) and “NetOGlyc 3.1” (http://www.cbs.dtu.dk/services/NetOGlyc-3.1/) for potential *N*-linked and *O*-linked glycosylation sites detection, respectively, and ProtScale^60^ (http://www.expasy.ch/cgi-bin/protscale.pl) for protein hydrophobicity–hydrophilicity plot.

### Phylogenetic analysis

Multiple sequence alignments and phylogenetic reconstructions were performed using the Phylogeny.fr platform (http://www.phylogeny.fr/)^61^. The deduced amino acid sequence of SfPER protein was independently compared with 38 complete and partial amino acid sequences corresponding to PM peritrophins identified in a *S. frugiperda* transcriptome^41^ and phylogenetic tree reconstructed using the maximum-likelihood method implemented in the PhyML program (vs. 3.0 aLRT)^62^ together with bootstrap analysis using 100 replicates. The graphical representation and edition of the phylogenetic tree were performed with TreeDyn vs. 198.3^63^.

### Analysis of *SfPER* transcript levels by RT-PCR

*SfPER* mRNA accumulation in different *S. frugiperda* life stages was determined by RT-PCR. Total RNA samples (1 μg) obtained as above, were used as templates for one-tube RT-PCR with the AccessQuick RT-PCR system (Promega). The *SfPER*-specific primers (GSP-F, forward) 5′-actgcgatccttctgaagctagac-3′ and (GSP-R, reverse) 5′-tccgggacaggacagagcaac-3′ were used in the reactions to amplify a 446-base pair (bp) fragment of the gene. A 150-bp internal amplification product from constitutive β-actin transcript produced with primers (Act-F, forward) 5′-cggtatcgtgctggactccggtg-3′ and (Act-R, reverse) 5′-gagtaacccctctcggtgaggatc-3′ was used as the control. Reaction products were visualized following electrophoresis in 2% agarose gels stained with GelRed (Biotium).

### Construction of expression vector

A 196-bp *SfPER* fragment spanning nt 149–344 and comprising the SfPER CBD1 region, referred to as SfPER-C1, was amplified by PCR using mutagenic oligonucleotides (forward) 5′-ggatcctctgaagctagacagatc-3′ and (reverse) 5′-aagctt**att**aaattggcctattgcc-3′, where underlined are mutagenic sequences containing restriction sites for BamHI and HindIII endonucleases, respectively. In reverse primer, an att sequence (in bold) was inserted to introduce two “TAA” stop codons in series in the final construct to increase efficiency of termination. The amplified product was BamHI/HindIII digested, purified and ligated into the same restriction sites of prokaryotic pSMT3 vector^64^ using standard molecular biology methods. Plasmid pSMT3 is a Small Ubiquitin-like Modifier (Sumo)/His-tag fusion system (from modified pET-28b expression plasmid). Recombinant pSMT3-SfPER-C1 clones were identified by colony PCR and confirmed by DNA sequence analysis (Macrogen). In the recombinant plasmid, SfPER-C1 was cloned in-frame with the 6xHis-SUMO sequence tag to produce a fusion 6xHis-SUMO/SfPER-C1 protein.

### Expression and purification of recombinant fusion 6xHis-SUMO/SfPER-C1 protein

Plasmid pSMT3-SfPER-C1 was transformed into *E. coli* BL21 (DE3) (Novagen) using the standard heat-shock method and grown in Luria Bertani (LB) medium supplemented with kanamycin at 50 μg/ml. Freshly synchronized-grown liquid cultures of recombinant bacteria were allowed to express the 6xHis-SUMO/SfPER-C1 protein by adding IPTG at a final concentration of 1 mM to the LB medium when OD_600_ reached approximately 0.4–0.6 (mid-log) and incubated at 37 °C with shaking for additional 4 h. The induced culture (50 ml) was then centrifuged (3000 × g, 10 min, 4 °C) and the cell pellet resuspended into 10 ml of lysis buffer (20 mM Tris-HCl, pH 7.7; 300 mM NaCl).

Cell lysis was achieved by 450 sonication pulses (400 W, 3 s with a 5 s interval) cooled in an ice water bath and suspensions were centrifuged at 13000 × g for 30 min at 4 °C. The clear supernatant was collected and passed through a 0.22 *μ*m filter and applied onto a 5 ml Chelating Sepharose TM Fast Flow resin (Amersham Pharmacia Biotech) column loaded with NiCl_2_. The 6xHis-SUMO/SfPER-C1 protein was purified using standard nickel affinity chromatography procedures. The resin was washed with five column volumes of washing buffer (20 mM Tris-HCl, pH 7.7; 300 mM NaCl; 20 mM imidazole). The protein was then collected from the column with elution buffer that contained the same components as in the washing buffer, except that the imidazole concentration was increased sequentially to 75, 150 and 300 mM. Samples (20 μl) taken at the elution peaks were boiled for 3 mins with 5 μl of 5x SDS sample buffer (0.3 M Tris-HCl, pH 6.8; 50% glycerol; 10% sodium dodecylsulfate; 0.125% bromophenol blue) supplemented with 5% β-mercaptoethanol and analyzed by reducing SDS-12% PAGE^65^. Proteins in the gels were visualized with Coomassie brilliant blue staining. The purified 6xHis-SUMO/SfPER-C1 protein was subjected to overnight dialysis at 4 °C against a new buffer (20 mM Tris-HCl, pH 8.0; 150 mM NaCl) to remove imidazole. Protein concentration was determined with a detergent-compatible formulation based on bicinchoninic acid (BCA) in the Pierce BCA Protein Assay Kit (Thermo Scientific), using bovine serum albumin (BSA) as the standard.

### Enzymatic digestion of 6xHis-SUMO/SfPER-C1 with SUMO protease

The cleavage of 6xHis-SUMO from SfPER-C1 in the fusion protein was performed with the SUMO protease Ulp1^66^. The Ulp1 protease was produced from a recombinant source, kindly provided in storage buffer (75 mM Tris, pH 8.0; 0.5 mM DTT; 5% glycerol, 1 mM EDTA) by A. Mussachio (CIGB, Havana, Cuba). First, the optimal amount of Ulp1 was determined by two-fold serial dilutions with a fixed amount of dialyzed 6xHis-SUMO/SfPER-C1 (20 μg) and starting with 10 units of Ulp1 in 100 μl reaction volume. Enzymatic reactions were incubated at 30 °C for 1 h and stopped by boiling for 3 min with 25 μl of 5X SDS sample buffer, supplemented with 5% β-mercaptoethanol. The reaction products were separated by reducing Tricine SDS-12.5% PAGE^67^. Low molecular weight polypeptides in tricine gels were visualized with Coomassie brilliant blue staining. Enzymatic reaction with optimal Ulp1 concentration was re-applied to the nickel column to which both SUMO and Ulp1 were bound through 6xHis tags, while most of SfPER-C1 protein eluted out in the flow-through (unbounded) fraction.

### Mass spectrometric analysis

The identity of 6xHis-SUMO/SfPER-C1 was confirmed by mass spectrometry at the CIGB Analytical Unit facilities, using a hybrid quadrupole-orthogonal time-of-flight mass spectrometer with a nanospray ion source (QTOF-2TM, Micromass). A mixture of tryptic peptides was loaded into the borosilicate nanoflow tips and submitted to 900 V and 35 V of capillary and cone voltage, respectively. The acquisition and processing of mass spectra were performed with MassLynx, v 4.0 (Waters). Protein RP-HPLC purity grade was 95% and identity confirmation was accomplished by comparison with the theoretical value for its amino acids sequence.

### Chitin affinity chromatography

Additional purification of 6xHis-SUMO/SfPER-C1 by chitin affinity chromatography was performed to confirm chitin binding. The protein (1 mg) was applied onto a chitin column (New England Biolabs) equilibrated with PBS 1X buffer (137 mM NaCl; 10 mM Na_2_HPO_4_; 1.8 mM KH_2_PO_4_; 2.7 mM KCl; pH 7.4). The column was fully washed with PBS 1X buffer before eluting the protein with elution buffer (20 mM Tris–HCl, pH 6.8; 5% SDS). The purification process was assessed by reducing SDS-12% PAGE as above

### Generation of polyclonal antibodies

The 6xHis-SUMO/SfPER-C1 protein was used as antigen to generate specific polyclonal antibodies in rabbits, provided by the National Centre for Laboratory Animals Breeding (CENPALAB) in Cuba. The immunization scheme was performed in strict accordance with the recommendations of the Ethics Committee of Experimental and Animal Welfare of the Laboratory Animals department at the Centre for Genetic Engineering and Biotechnology, which approved the protocol. Animals were immunized with 100 μg of the antigen mixed with Freund’s complete adjuvant (1:1, v/v) intramuscularly. Two further immunizations were carried at 15 day intervals but with Freund’s incomplete adjuvant. Harvested blood samples were centrifuged at 3000 × g for 15 min to isolate the serum, followed by application onto a Protein A-Sepharose resin in a XK-26 chromatographic column (Amersham Pharmacia Biotech) equilibrated with PBS 1X buffer (pH 8). Antibodies were eluted with 100 mM citric acid (pH 3). The pH of elution fractions was neutralized with 2 M Tris and buffer changed to (20 mM Tris, pH 7.6; 150 mM NaCl) using size exclusion chromatography with Sephadex G-25 (Amersham Pharmacia Biotech).

### Larval proteins extraction and analysis

Proteins from PM (anterior, middle and posterior) sections, fat body and cuticle, were extracted by mechanically grinding dissected tissues from 10 actively-feeding 6^th^-instar larvae with a pestle in extraction buffer (20 mM Tris, pH 6.8; 300 mM NaCl; 5% SDS). Protein concentration was determined with BCA Protein Assay Kit (Thermo Scientific) as above. Samples (20 μg) were boiled for 3 min with 5X SDS sample buffer without β-mercaptoethanol for separation by non-reducing SDS-12% PAGE.

### Western blotting assays

Electrophoresed proteins were blotted onto nitrocellulose membranes using a semi-dry transfer device (BIO-RAD). Proteins in the blots were visualized with 0.1% Ponceau S reversible staining to determine transfer efficiency. Membranes were blocked with 5% (w/v) non-fat milk powder in TBS (50 mM Tris, pH 7.6; 150 mM NaCl) for 30 min with constant shaking at RT, and then probed with a rabbit polyclonal anti-6xHis-SUMO/SfPER-C1 antibody, 1:25 dilution in TBS-T (TBS containing 0.2% Tween 20) for 1 h with constant shaking at RT. A monoclonal alkaline phosphatase (AP)-labeled goat anti-rabbit IgG (SIGMA) diluted 1:5000 in TBS-T was used for detection. Blots were washed four times with TBS-T, for 5 min each, and developed with 5-bromo-4-chloro-3-indolyl phosphate (BCIP)/nitro blue tetrazolium (NBT) insoluble substrate (SIGMA) in developing buffer (100 mM Tris, pH 9.5; 100 mM NaCl, 5 mM MgCl_2_).

### dsRNA synthesis

A 446-bp DNA template for *SfPER*-dsRNA synthesis was amplified via PCR using GSP-F and GSP-R primers elongated with T7 promoter sites. *In vitro* generation of dsRNA used a T7 RiboMAX Express RNAi System (Promega). Control experiments with non-specific dsRNA used a 611-bp fragment from the *E. coli β-D-glucuronidase* gene (GenBank accession no. CP001509) produced as previously described^36^.

### RNAi experiments

Newly-molted, 6 h-starved third-instar larvae were fed with a 0.25-μl drop of delivery buffer (DB: 10 mM Tris-Cl, pH 7.5; 10 mM EDTA; 10 mM sucrose) containing 2.5 μg dsRNA-SfPER (10 mg/ml). DB alone and DB containing dsRNA-Gus were used as controls. Replicates used 60 larvae for each treatment.

For *SfPER* gene suppression analysis, insects were dissected 48 h after droplet feeding. Total RNA from midgut pools of 12 larvae was isolated as above and used for One-step real-time RT-PCR with the aid of QuantiTect SYBR Green RT-PCR kit (QIAGEN) according to the manufacturer’s instructions. The 25 μl PCR reactions were performed in duplicates with 100 ng template RNA and 7.5pmol of each (SfPER-F, forward) 5′-cacacgaaatctgcaacaagttc-3′ and RACE-5′ primers that together amplify a 127-bp product in the *SfPER* sequence. The 150-bp β-actin amplicon produced with primers Act-F and Act-R was used for transcript normalization. A Rotor-Gene 3000 real-time cycler (Corbett Research) was used with the following program: a reverse transcription step for 30 min at 50 °C, a PCR initial activation step for 15 min at 95 °C (to activate the HotStarTaq DNA Polymerase), followed by 40 cycles of DNA denaturation for 15 sec at 95 °C, annealing for 30 sec (see Table S1 for annealing temperature for a specific primer set), and extension for 30 sec at 72 °C. The specificity of the PCRs was confirmed by melting curve analysis at 55–95 °C and all Ct values were obtained from two independent experiments. Amplification efficiencies were determined with the aid of standard curves (Table S1). The relative transcript levels were expressed as ‘Mean Normalized Expression’ data using Q-GENE software (http://www.gene-quantification.de/qgene.zip)^68^.

### Bt protoxin and bioassays

The Bt Cry1Ca1 protoxin (pCry1Ca1) produced in a recombinant Bt var. *israelensis* IPS-78/11 strain, carrying the plasmid pHY-1Ca, was obtained as described in Ayra-Pardo *et al*.^69^.

Insect growth inhibition (GI) bioassays in response to a 2 μg/cm^2^ concentration of pCry1Ca1 was performed on 24 larvae per RNAi treatment 48 h after droplet feeding as described in Rodriguez-Cabrera *et al*.^36^. Pre-weighed larvae were fed with artificial diet with or without pCry1Ca1 and re-weighed 48 h later. The percentage of GI was calculated as: %GI = [1 − (RGt/RGc)] × 100, where RGt and RGc represent the relative growth in the presence of toxin (RGt) and in the control (RGc) respectively. Relative growth (RG) was calculated as RG = [(W1 − W0)/W0], where W0 and W1 are the initial and final weight of the larva respectively^70^.

Larval and pupae weight, larval and pupae mortality, time of appearance of the first pupae and of adult emergence, occurrence of deformity in adult moths, and eggs viability were recorded and compared between RNAi treatments and controls.

### Data analysis

Data were analysed using the GraphPad Prism software vs. 4.00 for Windows. *SfPER* relative expression levels between RNAi-treated and control groups were compared using a *t* test (*p* ≤ 0.05). Analysis of variance (ANOVA) in conjunction with post-hoc Tukey–Kramer multiple comparisons test was performed to determine differences among treatments for insect performance upon feeding dsRNA, e.g. %GI, larval and pupae weight, % total emerged adults (*p* ≤ 0.05). The experiments were carried out at least twice with two independent replicates, and similar results were obtained. The standard error of means was used to compare the replicates^67^.

## Supplementary information

Functional expression of a peritrophin A-like SfPER protein is required for larval development in Spodoptera frugiperda (Lepidoptera: Noctuidae)
