# Supplementary material for: Functional expression of a peritrophin A-like SfPER protein is required for larval development in Spodoptera frugiperda (Lepidoptera: Noctuidae)
Source: Sci Rep. 2019 Feb 22;9:2630. doi: 10.1038/s41598-019-38734-0 (PMC6385298; doi:10.1038/s41598-019-38734-0)
Supplement: Supplementary file 1 — Functional expression of a peritrophin A-like SfPER protein is required for larval development in Spodoptera frugiperda (Lepidoptera: Noctuidae) [file 41598_2019_38734_MOESM1_ESM.pdf]

Functional expression of a peritrophin A-like SfPER protein is required for larval development in *Spodoptera frugiperda* (Lepidoptera: Noctuidae)

Claudia Rodríguez-de la Noval[1,♦,¶], Lianet Rodríguez-Cabrera[1,♦], Laurent Izquierdo[1], Luis A. Espinosa[2], Daily Hernandez[1], Milagro Ponce[1], Ivis Moran-Bertot[1], Pilar Tellez-Rodríguez[1], Orlando Borras-Hidalgo[3], Siliang Huang[4], Yunchao Kan[4], Denis J. Wright[5] and Camilo Ayra-Pardo[4,*]

Table S1. Primers and experimental conditions used in quantitative RT-PCR.

Figure S1. Pairwise sequence alignment between SfPER and SfPMP24. The amino acids sequence of SfPMP24 (171 amino acids) was obtained from Dias et al. (41).

Figure S2. Multiple sequence alignment of SfPER peritrophin A-like chitin-binding domains. In the alignment, each of the six conserved cysteines residues from consensus arrangement of peritrophin A domain (PAD) i.e. $C^1X_{13\text{-}18}$ $C^2X_5C^3X_{9\text{-}11}C^4X_{10\text{-}13}C^5X_{7\text{-}8}C^6$, has been enclosed in a square.

Table S1

Gene	Primer sequence (5’–3’)	Product size (bp)	Ta[a] (°C)	S[b]	E[c]	r[2d]
SfPER	*CACACGAAATCTGCAACAAGTTC*	127	58	-3.67	1.87	0.97
	CCGCAGACGACATTCTCGGGCCAGTC					
β-actin	*CGGTATCGTGCTGGACTCCGGTG*	150	58	-3.52	1.92	0.98
	GAGTAACCCCTCTCGGTGAGGATC					

[a]Annealing temperature in the PCR program for a specific primer set.

[b]Slope of the linear regression line of the amplification efficiency plot.

[c]Amplification efficiency calculated by Q-gene as: 10^(-1/slope).

[d]Reproducibility of the real time PCR reaction.

Figure S1

```
SfPMP24_Sf_2444.1   --------------------------------------------------   50
SfPER_MG786480      MKDTVLLLLCAVALAHSYVHNTQRCNCDPSEARQICESNYGLDDVLVAHE   50
                    1.......10........20........30........40........50

SfPMP24_Sf_2444.1   --------------------------------------------------   100
SfPER_MG786480      YCDKFYKCANGRPVAYQCPDNLLYDPVAERCEWPNEVNCGNRPISDGNDC   100
                    .........60........70........80........90.......100

                                                                ******
SfPMP24_Sf_2444.1   --------------------------------------------GSDGVL   150
SfPER_MG786480      NGTDVSSSTQEPVSSSSSSTTSRPIDDETCNCRPEEAPSICAADGSDGVL   150
                    .......110.......120.......130.......140.......150

                    ************ :************************************
SfPMP24_Sf_2444.1   VANENCNQFYICVYGKPVALSCPGNLLYNPYIEQCDWPENVDCGDRVVPD   200
SfPER_MG786480      VANENCNQFYICDHGKPVALSCPGNLLYNPYIEQCDWPENVDCGDRVVPD   200
                    .......160.......170.......180.......190.......200

                    ***************************:**********************
SfPMP24_Sf_2444.1   PSQTPPPGPGSSPNPTPSPTASTPSSGTCNCRPEEAPSICAADGSDGVLV   250
SfPER_MG786480      PSQTPPPGPGSSPNPTPSPTASTPSSGACNCRPEEAPSICAADGSDGVLV   250
                    .......210.......220.......230.......240.......250

                    **************************************************
SfPMP24_Sf_2444.1   AHEICNKFYKCNHGKPVSLYCYGNLLYNPYIEQCDWPENVVCGQRVVPDP   300
SfPER_MG786480      AHEICNKFYKCNHGKPVSLYCYGNLLYNPYIEQCDWPENVVCGQRVVPDP   300
                    .......260.......270.......280.......290.......300

                    * ************
SfPMP24_Sf_2444.1   GQTRRPGSSQDHRF   314
SfPER_MG786480      GPTRRPGSSQDHRF   314
                    .......310....
```

Figure S2

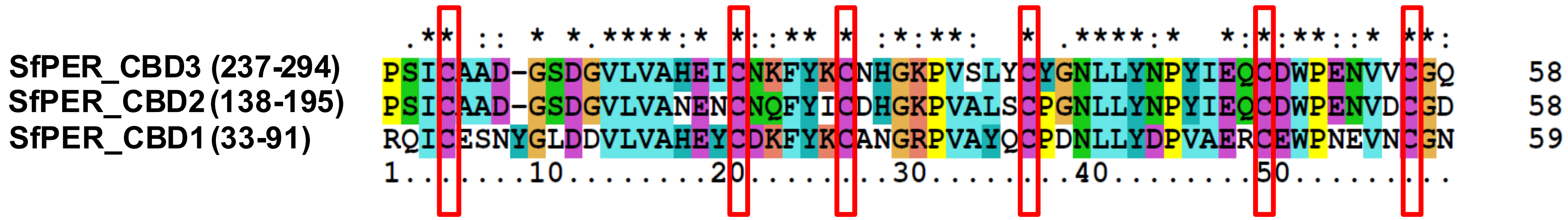
SfPER_CBD3 (237-294)
PSICAAD-GSDGVLVAHEICNKFYKCNHGKPVSLYCYGNLLYNPYIEQCDWPENVVCGQ
58
SfPER_CBD2 (138-195)
PSICAAD-GSDGVLVANENCNQFYICDHGKPVALSCPGNLLYNPYIEQCDWPENVDCGD
58
SfPER_CBD1 (33-91)
RQICESNYGLDDVLVAHEYCDKFYKCANGRPVAYQCPDNLLYDPVAERCEWPNEVNCGN
59
1.........10.........20.........30.........40.........50.........
